# Valproate reopens critical-period learning of absolute pitch

**DOI:** 10.3389/fnsys.2013.00102

**Published:** 2013-12-03

**Authors:** Judit Gervain, Bradley W. Vines, Lawrence M. Chen, Rubo J. Seo, Takao K. Hensch, Janet F. Werker, Allan H. Young

**Affiliations:** ^1^Laboratoire Psychologie de la Perception, CNRSParis, France; ^2^Laboratoire Psychologie de la Perception, Université Paris Descartes, Sorbonne Paris CitéParis, France; ^3^Department of Psychiatry, Institute of Mental Health, University of British ColumbiaVancouver, BC, Canada; ^4^Department of Linguistics, University of MarylandCollege Park, MD, USA; ^5^School of Medicine, University of QueenslandBrisbane, QLD, Australia; ^6^Department of Molecular Cellular Biology, Center for Brain Science, Harvard UniversityCambridge, MA, USA; ^7^Department of Psychology, University of British ColumbiaVancouver, BC, Canada; ^8^Centre for Affective Disorders, Institute of PsychiatryKing's College London, UK

**Keywords:** critical period reopening, learning, absolute pitch, valproate, histone-deacetylase inhibitors, human adults

## Abstract

Absolute pitch, the ability to identify or produce the pitch of a sound without a reference point, has a critical period, i.e., it can only be acquired early in life. However, research has shown that histone-deacetylase inhibitors (HDAC inhibitors) enable adult mice to establish perceptual preferences that are otherwise impossible to acquire after youth. In humans, we found that adult men who took valproate (VPA) (a HDAC inhibitor) learned to identify pitch significantly better than those taking placebo—evidence that VPA facilitated critical-period learning in the adult human brain. Importantly, this result was not due to a general change in cognitive function, but rather a specific effect on a sensory task associated with a critical-period.

## Introduction

Absolute pitch (AP), the ability to identify or produce the pitch of a musical sound without any reference point, has long fascinated musicians, music scholars, psychologists, and neuroscientists (Stumpf, 1883; Mull, 1925; Takeuchi and Hulse, 1993; Zatorre, 2003; Levitin and Rogers, 2005). Individuals who possess AP, constituting about 0.01% of the general population, are able to identify the pitch class, i.e., one of the 12 notes of the Western musical system, e.g., C, D, G#, of a sound with great accuracy (varying between 70–99%, depending on the task, as compared to 10–40% for non-AP individuals, Takeuchi and Hulse, 1993). Their errors are usually not more than a semitone away from the target sound, as compared to 3 or more semitones for non-AP individuals. AP possessors also make octave errors, i.e., they identify the pitch class, but not the pitch height correctly, labeling a C_4_ (middle C) as C_5_, an octave higher. Pitch class and pitch height identification are thus believed to be separate processes, and only the former constitutes a crucial test for AP (Takeuchi and Hulse, 1993; Levitin and Rogers, 2005), which AP possessors perform effortlessly and automatically. Their reaction times are faster, at least when responding correctly, than those of non-possessors (Miyazaki, 1990), suggesting that the former have direct access to pitch names in memory, whereas the latter might rely on relative pitch to calculate pitch class. In light of AP possessors' highly accurate and automatic identification of pitch class, it has been suggested that in addition to perceptual mechanisms, AP involves the association of (verbal) labels for pitch classes in long-term memory (Zatorre, 2003; Levitin and Rogers, 2005).

The central role of these associations is further supported by functional imaging studies showing the involvement of the left posterior dorsolateral frontal cortex and the planum temporale, known to be responsible for learning conditional associations (Zatorre et al., 1998; Ohnishi et al., 2001; Bermudez and Zatorre, 2005; Wilson et al., 2009). In addition, AP possessors have a larger planum temporale (Zatorre et al., 1998), with a left-right asymmetry involving a smaller right planum temporale and an increased leftward asymmetry in AP possessors (Keenan et al., 2001), and show hyperconnectivity in the temporal cortex (Loui et al., 2011), facilitating tone-label mapping.

Importantly, acquiring AP has a critical period (Levitin and Zatorre, 2003; Russo et al., 2003). A critical period is a fixed window of time, usually early in an organism's lifespan, during which experience has lasting effects on the development of brain function and behavior. The principles of critical period phenomena and neural plasticity are increasingly well understood both at the behavioral/experiential (Kleim and Jones, 2008) and at the molecular/cellular level (Hensch, 2005). Specifically, behaviorally induced plasticity in the healthy brain, typically after the end of the relevant critical period, can lead to improvement beyond normal or average performance levels. However, for many tasks, this requires targeted training—simple routine use is often insufficient. The factors known to influence the efficiency of such targeted training include the number of repetitions involved, the intensity of the training as well as the relevance or saliency of the stimuli or task trained. Importantly, such training-induced learning is quite specific to the trained task and to the underlying brain networks, although some transfer to other, related domains of knowledge or skills is sometimes possible. At the cellular level, critical periods close when maturational processes and experiential events converge to cause neuoro-physiological and molecular changes that dampen or eliminate the potential for further change (Hensch, 2005; Bavelier et al., 2010), thus imposing “brakes” on neuroplasticity. One of the epigenetic changes leading to decreased plasticity after the critical period involves the action of HDAC, an enzyme that acts as an epigenetic “brake” on critical-period learning (Morishita and Hensch, 2008; Qing et al., 2008). Research has shown that inhibition of HDAC can reopen critical-period neuroplasticity in adult mice to enable recovery from amblyopia (Putignano et al., 2007; Silingardi et al., 2010), and to facilitate new forms of auditory learning (Yang et al., 2012).

The age of onset of musical training has been shown to predict AP acquisition (Deutsch et al., 2009), indicating the presence of a critical period. AP is most typically seen in individuals who started musical training before 6 years of age (Levitin and Zatorre, 2003; Russo et al., 2003; Miyazaki and Ogawa, 2006). Indeed, the distribution of the age of first formal musical training in a large number of individuals with AP can be modeled with a gamma function with a mode at 4–6 years (Levitin and Zatorre, 2003). Training that begins after the age of 9 very rarely leads to AP, and there are no known cases of an adult successfully acquiring it (Brady, 1970; Ward and Burns, 1999; Levitin and Rogers, 2005). The appropriate type of input, i.e., training associating absolute pitches to labels, thus needs to be available before the end of the critical period for AP to develop. For most individuals, this is not the case, as the two major sources of auditory input during early development, language, and the Western musical tradition mainly rely on relative pitch, explaining why not all musically trained individuals have AP. In the absence of AP cues during the critical period, the perceptual system is reorganized, shifting weight from absolute to relative pitch information (Takeuchi and Hulse, 1993; Saffran and Griepentrog, 2001; Saffran, 2003, although see Trehub, 2003 for a somewhat different view).

AP is thus particularly interesting from a neuro-scientific perspective, as it provides a model for understanding the interaction of genes and experience on the development of neural and cognitive function (Zatorre, 2003). In the current study, we explored whether a reopening of the critical period was possible for AP learning in human adults. We sought to establish whether the administration of valproate (VPA), a commonly used anticonvulsant and mood stabilizer, known to inhibit HDAC and modulate the epigenome to promote neuroplasticity (Phiel et al., 2001; Schloesser et al., 2007; Machado-Vieira et al., 2011) would facilitate training naïve, non-musician adults on the identification of pitch classes in a classical AP task (Deutsch et al., 2006).

Previous studies (Meyer, 1899; Mull, 1925; Wedell, 1934; Hartman, 1954; Lundin, 1963; Cuddy, 1968; Russo et al., 2003) have shown that training improves adults' AP abilities only under restricted conditions. Three factors appear to play a particularly important role: participant's previous musical training/experience, whether a single tone or a series of tones are used for training, and the duration/intensity of the training. Notable improvement is achieved only when musically highly proficient participants are trained on a single note for extensive periods (from several weeks to several months) and tested on the recognition of this single target tone among several non-target ones (Mull, 1925; Cuddy, 1968). It is possible, however, that at least in some such cases, the improvement is actually due to greater familiarity with the particular task, tone series or procedure, rather than to a genuine increase in AP ability (Takeuchi and Hulse, 1993). Improvement is much more limited when musically untrained participants are tested (Cuddy, 1968), when participants are trained on a series of pitch classes rather than on just one pitch class (Cuddy, 1968), or when training is relatively short or less intense (Vianello and Evans, 1968). Prior musical training is particularly important, as musically proficient participants typically perform better than chance (Mull, 1925; Lundin, 1963) and better than musically naïve participants (Cuddy, 1968) on pitch identification already prior to AP training, it is thus not surprising that some AP improvement may be achieved in this population. A recent finding further shows that in addition to early musical training, the current musical environment also contributes to (the maintenance of) AP abilities, suggesting the presence of some residual plasticity for AP, at least in individuals in whom AP emerged during the early critical period (Wilson et al., 2012).

The use of a single tone vs. a series of tones for training is also highly relevant, as the two training methods might tap into different underlying pitch perception abilities or might represent tasks of varying degrees of complexity. In our current understanding, the identification of only one pitch class serving as an internal reference, sometimes referred to as quasi-AP or single tone AP, is qualitatively different from true AP, whereby the individual is able to identify a large number of pitch classes automatically and without an internal or external reference. Albeit often similar in the percentage of correct identifications, the two types of abilities can be distinguished on the basis of reaction times, as AP possessors have faster reaction times than absolute tuning tone processors (Miyazaki, 1990; Levitin and Rogers, 2005). It needs to be noted, however, that according to some authors (e.g., Cuddy, 1968) the single tone method might also lead to true AP eventually.

The length and intensity of training also affects performance (Brady, 1970). Fast improvement is observed for tones that are separated by large pitch distances, whereas more extended training is necessary for pitches that are closer together (Hartman, 1954). Not surprisingly, the latter are in general harder to learn and to discriminate than the former, with musically naïve participants not being able to achieve discrimination errors smaller than about 5 semitones with pitch classes that are separated by small distances (Wedell, 1934). Further, interference effects are sometimes observed when tone series are taught gradually, involving the introduction of new tones that fall in between already trained ones, decreasing pitch distance, and disrupting the subjective organization of the scale.

To assess whether taking VPA could reopen the opportunity for critical-period-like learning in adults, we conducted a randomized, double-blind, placebo-controlled study, in which 24 young adult males received either placebo or VPA treatment in a cross-over design with two treatment arms. Participants underwent 15 days of treatment during which they took capsules (VPA or placebo) each day. During the second week of treatment, participants observed training videos that taught participants to associate six pitch classes from the 12-tone Western musical system (e.g., C, D, E, F#/Gb, G#/Ab, A#/Bb) with six proper names (e.g., Sarah, David, Francine, Jimmy, Karen, Leo). We chose to use proper names instead of the actual note names to make the task equally novel and accessible for participants with and without any prior musical training, and to divert attention from the music theoretical aspect of the task. We acknowledge that this may interfere with existing knowledge of actual musical note names in the participants who had sufficient musical training, but given that there were few such participants in our study, we considered that the advantages of using proper names outweighed the potential negative influence of such interference. On day 15, participants were given a post-treatment assessment for AP in which they heard 18 synthesized piano tones and had to identify the proper name associated with the pitch class of each tone. After the first treatment arm, a washout period of 2 to 4 weeks elapsed. Eighteen out of the 24 original participants then entered the second treatment arm, which was similar to the first one, except that the drug (VPA or placebo) not received during the first arm was administered.

Given the difficulty of improving AP performance in adulthood, we hypothesize that in our task, even a small advantage in pitch class identification in the VPA as compared to the placebo group is suggestive of the reopening of plasticity, as musically naïve participants were trained for a relatively short time period on several pitch classes, conditions under which no existing study has shown any improvement in AP. The strong hypothesis is that there might be improvement in the VPA condition in both arms of the design. However, since new training is introduced in the second treatment arm, successful learning in the first arm might carry over to and interfere with learning in the second arm. Effects are thus more likely in the first arm only. This study is, therefore, intended as a proof-of-concept demonstration that critical-period-like AP learning may be at least partly restored by using a drug to remove the epigenetic brakes on neural plasticity.

## Materials and methods

### Participants

The twenty four participants who took part in the study were healthy, right-handed, monolingual, English-speaking adult males (median age = 23, range = 18–27). A software malfunction corrupted the data for one participant in the first treatment arm, leaving 11 who took VPA, and 12 who took placebo. Participants gave informed, written consent following the protocol approved by the University of British Columbia clinical ethics review board and Health Canada. Exclusion criteria for the study included taking any medication with psychoactive effects, recreational drug use in the 6 months prior to participation, drug or alcohol abuse, being functionally bilingual or studying a second language at the university level, having perfect pitch or AP for musical tones, and being involved in an occupation requiring a high level of vigilance. Only males were included in the study as a caution for potential health risks that VPA might have on pregnant women.

Participants did not report having complete, partial or quasi AP, and have received no or little musical training (mean 2.4 years). Importantly, those who had received any musical training all started after the age of 7 years, some as late as 17 years (with a mean and a median of 12 years), well beyond the critical age of 4–6 years (Levitin and Zatorre, 2003). It is, therefore, unlikely that there were AP possessors among our participants.

During a screening session, we confirmed each participant's suitable health by means of a medical examination with a physician, which included a medical history. We also collected a blood sample to check for normal levels of hepatic enzymes, platelets, amylase, and ammonia. These as well as participants' psychological state and mood were also monitored throughout the entire duration of the study to screen for potential adverse effects. The participants completed questionnaires at the screening session about demographics, music experience, language training, and handedness, as well as an assessment of IQ with the North American Adult Reading Test (NAART). Table 1 summarizes these data.

**Table 1:** **Participants' data from the screening assessment**.

	**Mean (*SD*)**	**Range**	**Median**
Age (years)	22.6 (2.6)	18–27	23
IQ (FSIQ)	110 (6.8)	97–122	110
Handedness^*^	86.3 (16.1)	37–100	89
Time spent learning a second language (years)	2 (2.1)	0–8	2
Proficiency on a second language^**^	1.1 (0.86)	0–3	1
Number of years of music practice	2.4 (3)	0–10	1
Age the music training began (years)^***^	12 (2.5)	7–17	12

* *The Edinburgh Handedness Inventory scale ranges from −100 (fully left-handed) to +100 (fully right-handed)*.

** *Proficiency on a second language was based on a self-rating scale from 0 to 5, with 5 being very proficient*.

*** *These statistics are based upon the 14 participants who had at least some musical practice experience*.

We asked participants to maintain normal patterns of consumption of caffeinated beverages over the course of the study. At the end of their participation, we remunerated the participants for the time they devoted to the study at a rate of $10 per hour. The maximum remuneration for participants who completed the study came to $270.

Of the twenty four participants, 18 completed the second treatment arm. The six participants who only completed the first treatment arm either had to leave the study or had to be removed partway through for the following reasons: scheduling conflicts; travel; concern that side effects during the second treatment session might interfere with performance at a job interview; suffering a concussion due to an unrelated accident during the second treatment arm; loss of contact during the washout period with no explanation. No participant reported leaving the study because of any actual side effects from the treatment.

## Material

### Training

The training video taught participants to associate six pitch classes from the 12-tone Western musical system (e.g., C, D, E, F#/Gb, G#/Ab, A#/Bb) with six proper names (e.g., Sarah, David, Francine, Jimmy, Karen, Leo). Each pitch class was presented in three consecutive octaves using synthesized piano tones. During the training, a name would appear on the screen while the subject heard examples of the corresponding pitch class. The training for the second treatment arm involved six pitch classes and six proper names that were not used in the first arm of the study. Thus, for the two treatment arms in total, we used all 12 pitch classes of the Western musical system, and presented them in three consecutive octaves, based on Deutsch et al.'s study (2006).

The training videos included three blocks. Each block provided an opportunity for the participants to associate the six names with the corresponding pitch classes. During the first block, a subject saw one of the six names on the screen, and heard in succession the three examples of the pitch class corresponding to that name. The examples, which were piano tones of the same pitch class in three consecutive octaves, occurred one after another from the lowest octave to the highest. The second block was identical to the first, except that the ordering for the octaves was scrambled, such that the piano tones corresponding to a name did not necessarily occur from lowest to highest. In the third block, the stimuli occurred in a semi-random order, one at a time, and paired with the corresponding name. The semi-randomization was unique, and followed the same rules as in the task (see below). The length of the training was based on the duration of the VPA regimen employed in previous studies using VPA with healthy participants (Bell et al., 2005a,b). Specifically, participants trained for 7 days (from the 8th to the 14th day of the 14-day-long regimen), i.e., starting once the full dose of VPA was reached.

### Test

**Figure 1:**
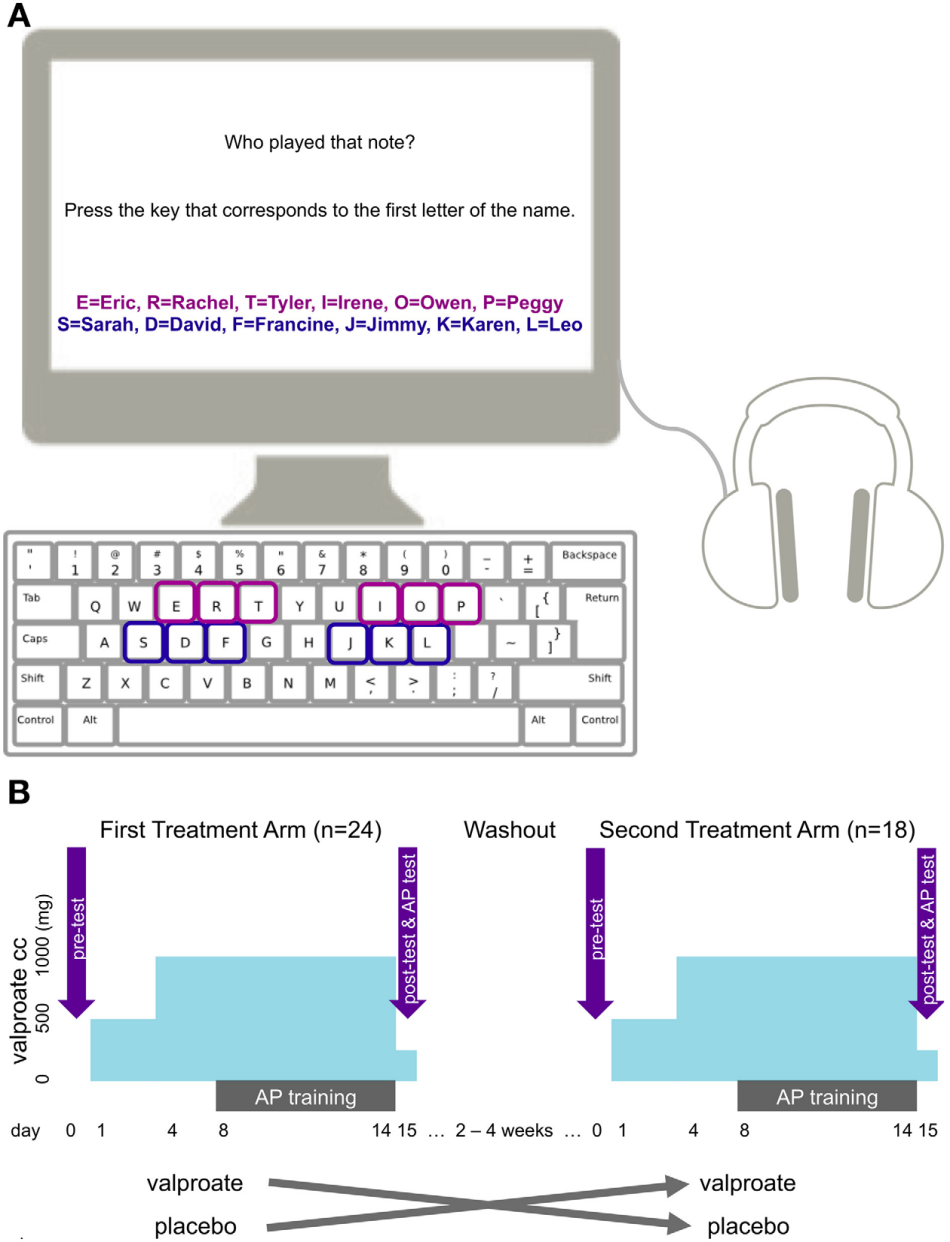
**(A)** The setup of the AP task. The purple characters and squares indicate names and corresponding keys used in one treatment arm, the blue ones indicate those used in the other treatment arm. Colors appear here for illustration purposes only. Only black characters were used in the actual experiment and response keys were not highlighted. **(B)** The cross-over design of the study with the two treatment arms, VPA regimen, training and test times.

During test, the six proper names appeared in a horizontal row on the screen in the same order for every subject (Figure 1A). Participants heard one synthesized piano tone per trial, for a total of 18 trials, in a semi-randomized order, the only constraint being that successive tones were separated by an interval greater than one octave. The tones were 500 ms in duration and interleaved by 3750 ms of silence, and were identical to the stimuli used during training. During the 3750ms period following each tone, participants had to identify the proper name associated with the pitch class of the tone by pressing the keyboard key corresponding to the first letter of the associated name (Figure 1A). The maximum score for the task was 18, the minimum 0, and chance performance corresponded to a score of 3.

Our stimuli were synthesized piano tones as in Deutsch et al.'s (2006) study. Unlike pure tones, stimuli generated by (synthesized) musical instruments contain cues other than frequency to pitch class identity, e.g., timbre. Intermediate and poor AP possessors are known to perform considerably worse with pure tones than with instrument-generated sounds (Lockhead and Byrd, 1981; Bermudez and Zatorre, 2009). Since we only expected a moderate improvement in AP perception in our study, we decided to avoid using pure tones.

This procedure was similar to that used by Deutsch and colleagues (Deutsch et al., 2006). However, it differed in two primary ways: (1) We divided the total set of test tones (36 in total) in half. Doing this enabled us to use an equal number of notes in the two task versions for participants who completed both treatment arms. As a result, we had half as many trials (18 in total) as Deutsch and colleagues. (2) Our participants learned associations between common names and the test tones, whereas Deutsch and colleagues used musical note names.

The names were common first names, the first letters of which appeared on the same row of the keyboard (e.g., s, d, f, j, k, l). Half of the names were female, and the other half male. For one treatment arm, the names were Sarah, David, Francine, Jimmy, Karen, and Leo. For the other, Eric, Rachel, Tyler, Irene, Owen, and Peggy. All of the names were bisyllabic and started with a different letter than the actual name of the musical note with which they were associated. The pitch classes in each treatment arm formed a whole-tone scale by selecting every other pitch from the 12-tone keyboard. For one treatment arm, the pitch classes were thus A#/Bb, C, D, E, F#/Gb, G#/Ab, for the other A, B, C#/Db, D#/Eb, F, G.

At the beginning of the task, there were four practice trials allowing participants to get used to the name—response key associations. During each practice trial, participants heard one of the common names spoken over the headphones, and then had to press the first letter of the name on the keyboard. We synthesized the names using an American English female voice in MBROLA (Dutoit, 1997). Participants only heard these synthesized names during the practice trials.

The AP task was administered in the post-treatment assessment only, because the participants had to learn novel associations between the proper names and the musical notes during training.

### Procedure

We used a randomized, double-blind, placebo-controlled design with a crossover between the two treatment arms (Figure 1B). We randomly counterbalanced participants into two treatment groups (VPA and placebo) in counterbalanced blocks of four. We used random allocation software to produce the randomization (Saghaei, 2004). The participants, experimenters, and raters were blind to treatment conditions. The eighteen participants who completed a second treatment arm crossed over into the treatment condition they had not undergone during their first treatment regimen. That is, a subject who received placebo first took VPA for the second treatment arm, and vice versa.

Each treatment arm comprised two assessments, one pre-treatment, and one post-treatment, separated by a two-week treatment period. The pre-treatment assessment consisted of blood samples and tests of mood and cognitive abilities^1^. No AP assessment could be administered at this point, as participants, not having considerable musical training, did not necessarily know the names of musical notes. On day 15, participants returned for a post-treatment session that was equivalent to the pre-treatment session, with the addition of a test for AP. There was a washout period of at least 2 weeks and no more than 4 weeks between the first and second treatment arms. The participants began taking capsules on the morning after the pre-treatment assessment. For participants in the VPA condition, the regimen included taking 500 mg (two 250 mg capsules, one in the morning and one at night) for 3 days (days 1–3), followed by 1000 mg (four 250 mg capsules, one in the morning, one in the afternoon, and two at night) for 11 days (days 4–14), and taking 250 mg (one capsule) on the morning of the post-treatment assessment (day 15).

The design of the treatment regimen was based upon previous studies (Bell et al., 2005a,b), and complied with Health Canada guidelines for administering VPA. The placebo regimen was identical to the VPA regimen and the placebo capsules were matched with the VPA capsules in terms of weight and appearance.

On days 8–14 of each treatment, we instructed participants to undergo an on-line training program for approximately 10 min per day. During each online training session, they observed a video, which trained associations between piano tones and proper names. After each video, the website prompted participants to answer a question about the content of the training video in order to gauge quality of attention (e.g., “How many women played notes in the video?,” “How many notes did each person play?” etc.). The website also recorded information about the start time and duration of the training.

Participants who completed both treatment arms filled out a form in which we asked them to guess during which treatment arm we had administered the VPA, and to report any symptoms they experienced while taking the capsules. Every laboratory session included a blood sample collection and assessments of mood and cognitive ability. We assessed mood using the Visual Analog Scales (VAS) for mood, the Beck Depression Inventory (BDI-II), and the Altman Self-Rating Scale for Mania (ASRM).

The cognitive assessment included the Ruff Neurobehavioral Inventory (RNBI-24), Rey Auditory Verbal Learning Test (RAVLT), Stroop Task, and Digit Span Test. We also assessed depth perception with the RANDOT Pre-school Stereoacuity Test, and visual acuity with the Regan Acuity Test. We used different word lists of the RAVLT for the first and second treatment arms. In addition to using them as experimental variables, we also monitored the results of the BDI-II and the RNBI-24 to identify any adverse effects of taking VPA on mood and cognition (e.g., suicidal thoughts or confusion). We did not find any adverse effects in any of the participants.

To assess AP, we used a computerized task programmed in PsyScope X Build 55 (http://psy.ck.sissa.it/). Participants worked alone in a room, and followed instructions on the computer screen about how to respond during the task by pressing keys on the computer keyboard. An experimenter introduced the task, and returned to the testing room after the completion of the task to record any comments. We used different stimulus sets for the two treatment arms in order to minimize learning and carry-over effects.

## Results

### First treatment arm

**Figure 2:**
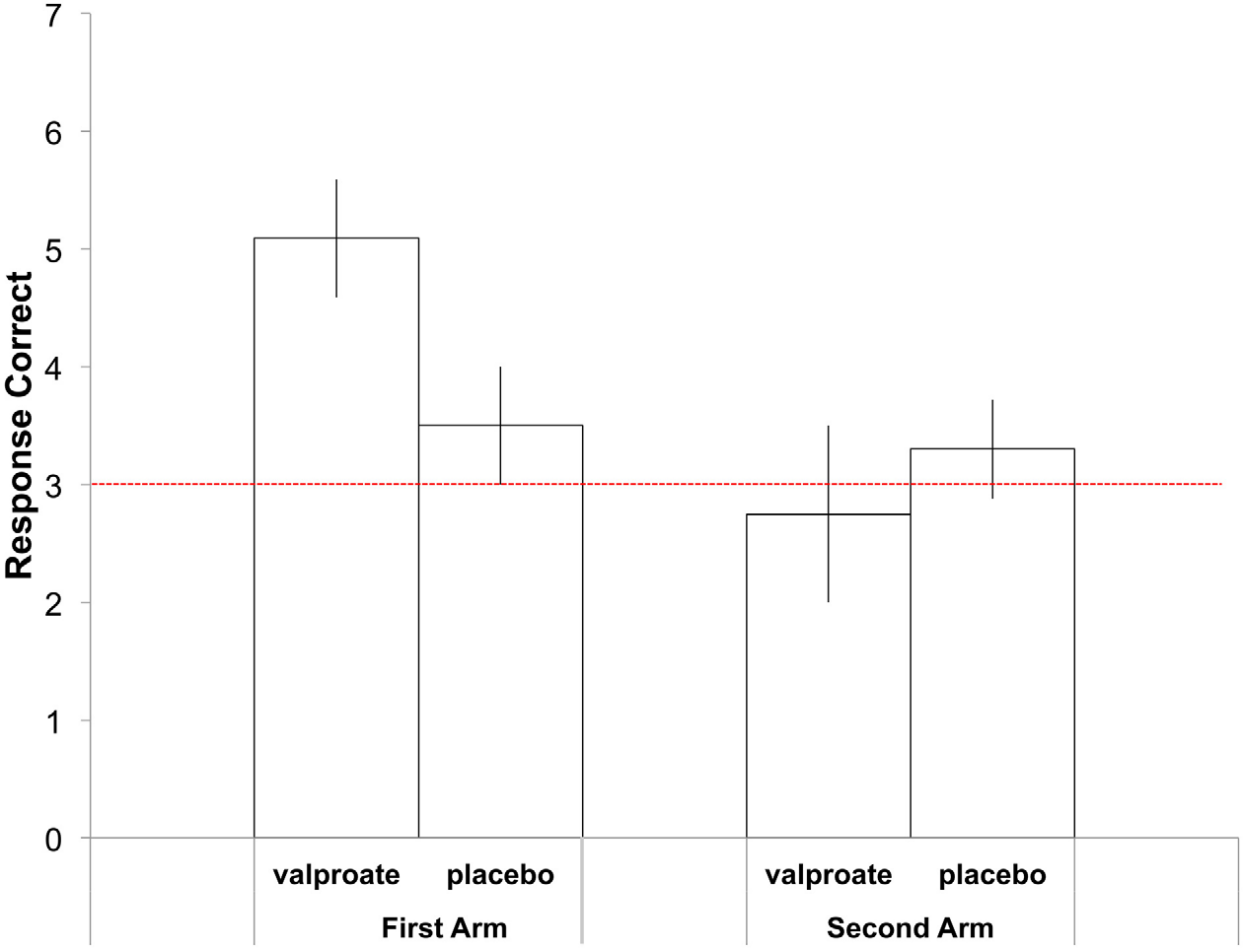
**Average number of correct responses in the AP task in the first (left) and second (right) treatment arm**. Errors bars indicate the standard error of the mean. The dashed red line indicates chance performance.

**Figure 3:**
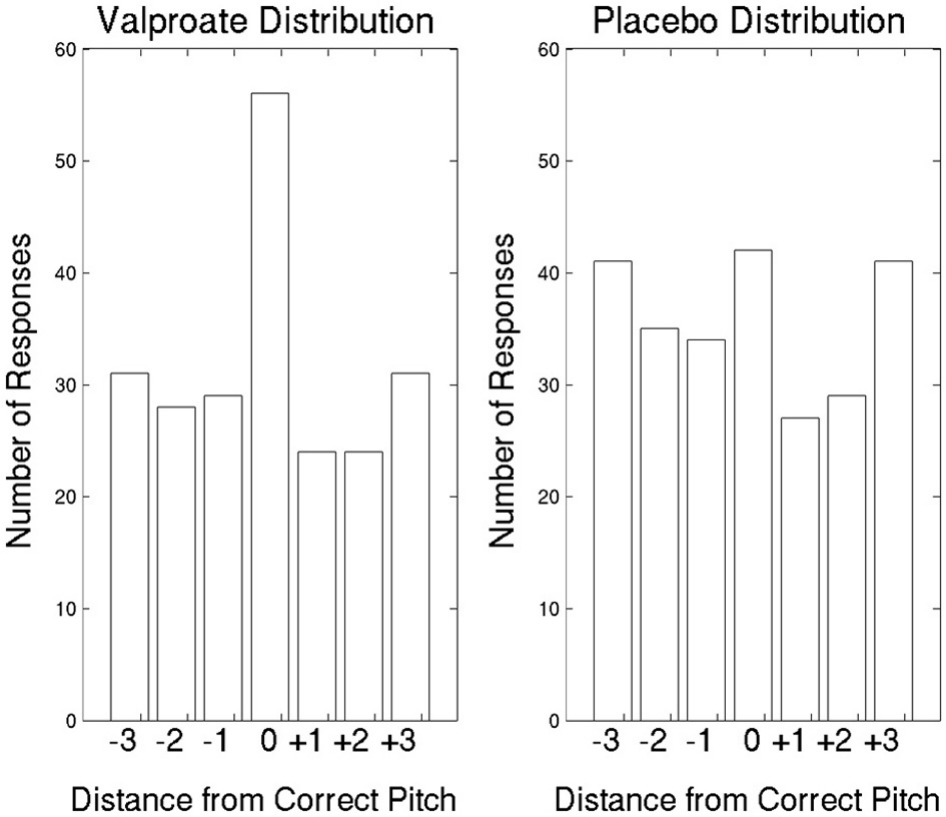
**The AP data in the first treatment arm, with errors shown as deviations from the correct pitch category measured in whole tones**. Pitch categories repeat every octave. Thus, +3 whole tones is the same as −3 whole tones, and the corresponding bars in the figure represent the same responses. The expected correct response is a distribution with a mode at 0, with a deviation of 0.

In the first treatment arm, the average correct responses were 5.09 in the VPA group and 3.50 in the placebo group (Figure 2). Participants^2^ in the VPA condition performed significantly above chance, which was 18/6 = 3 [*t*_(10)_ = 4.08, *p* = 0.002], whereas those in the placebo condition performed at chance level [*t*_(11)_ = 1.32, *p* = 0.21]. To further probe performance, we plotted the data with errors shown as deviations from the correct pitch category measured in whole tones (Figure 3). The distribution of the placebo group is flatter than that of the VPA group, indicating greater randomness. In addition, errors appear to have been random in the VPA condition, though participants were correct more often. This suggests that learning was absolute in the sense that the structure of the categories participants formed did not represent nearness from one category to another, strengthening the finding.

In a One-Way ANOVA with Condition (VPA/placebo) as a between-subject factor, we obtained a significant effect of Condition [*F*_(1, 21)_ = 6.37, *p* = 0.02] due to better performance in the VPA group compared to the placebo group.

To test whether this positive effect of VPA was specific to AP perception, or whether it resulted from a general change in mood and/or cognition, we conducted ANOVAs with Time (pre-treatment/post-treatment) as a within-subject and Treatment (VPA/placebo) as a between-subject factor on the measures of mood and/or cognition (with the exception of the RANDOT Stereoacuity test, which yields ordinal data and was thus entered into the non-parametric Friedman test). A significant Time X Treatment interaction would indicate a possible differential effect of VPA compared to placebo. No such interaction was obtained for any of the measures. Table 2 summarizes the scores of the measures for which a significant main effect was obtained.

**Table 2:** **Analysis of mood and cognitive measures in the first treatment arm**.

**Measure**	**VPA**		**Placebo**		**Main effects**	**Inter-actions**
	**Pre**	**Post**	**Pre**	**Post**		
**MEASURES OF MOOD**						
VAS withdrawn/sociable	6.01	6.00	7.13	5.25	*Time*: pre > post	
					*F*_(1, 22)_ = 4.36, *p* = 0.049	
VAS depression	0.58	1.45	1.33	2.36	*Time*: pre < post	
					*F*_(1, 22)_ = 7.71, *p* = 0.01	
VAS irritable/peaceful	6.76	5.31	5.53	5.39	*Time*: pre > post	
					*F*_(1, 22)_ = 4.63, *p* = 0.04	
**MEASURES OF COGNITION**						
VAS mentally slow/Quick-witted	6.82	6.03	4.78	4.99	*Treatment*: VPA > plac	
					*F*_(1, 22)_ = 4.38, *p* = 0.048	
RAVLT items 1–5	55.16	53.5	59.42	71.41	*Time*: pre < post	
					*F*_(1, 22)_ = 4.82, *p* = 0.04	
					*Treatment*: VPA < plac	
					*F*_(1, 22)_ = 4.38, *p* = 0.048	

For the participants in the VPA condition, the average blood concentration of VPA at the post-treatment assessment was 567 μmol/L (range: 261–854, *SD* = 165.53). The active range of VPA is considered 350–700 μmol/L. The concentration for one subject fell below this range (261 μmol/L), and the concentrations for three other participants fell above this range (708, 732, 854 μmol/L). The concentrations for the remaining eight participants fell within the typical active range. Participants' performance on the AP task did not show a significant correlation with VPA levels in the blood (*r* =.36, *n* = 11, *p* = 0.28).

We also calculated the number of training sessions each subject completed. We counted a training session as complete if the subject both watched the full length of the video (up to within 15 s of the end) and answered the subsequent test question correctly. Based on these criteria, participants completed an average of 4.63 AP training sessions (*SD =* 2.06, range: 0–7). Notably, 0 training sessions did not signify that a subject did no training; all participants did train for this task online. Participants who *completed* 0 training sessions either systematically stopped the video partway through, or watched the entire length and then failed to answer the question at the end correctly. There was no significant correlation between the number of completed training sessions and performance (*r* = 0.13, *n* = 23, *p* = 0.55).

We also ran a correlation analysis to compare AP performance in this treatment arm with the number of years of musical training each subject had completed (*r* = −0.12, *n* = 23, *p* = 0.60), and the age of start of musical training for those participants who did have musical training (*r* = −0.20, *n* = 14, *p* = 0.50), but neither reached significance. Importantly, our participants were musically naïve, had little musical training, and all started music after age 7 with a mean age of 12, i.e., after the critical period. Thus, the absence of any correlation between AP performance in our study and participants' musical training is not unexpected.

### Second treatment arm

In the second treatment arm, the average correct responses were 2.75 in the VPA group and 3.33 in the placebo group (Figure 2). Participants performed at chance level in both groups [VPA: *t*_(7)_ = 0.333, ns., placebo: *t*_(9)_ = 0.709, ns.]. In a One-Way ANOVA with Condition (VPA/placebo) as a between-subject factor, no difference was found between the two groups [*F*_(1, 16)_ = 0.452 ns.].

### Crossover

**Figure 4:**
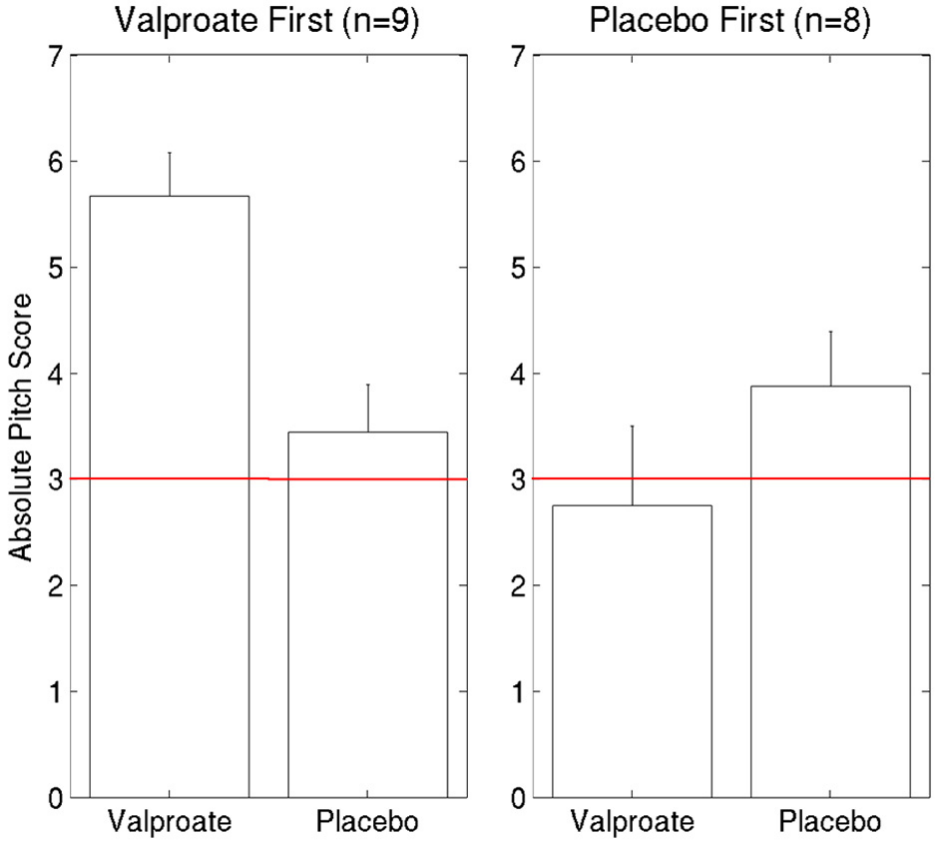
**Comparing the effects of VPA and placebo for each treatment order**. The red line indicates chance performance.

To compare the two treatment arms for the 18 participants who completed the whole study (9 received VPA first, 8 placebo first and as mentioned before, 1 participant's data was corrupted by a computer error), we also ran an ANOVA with Condition (VPA/placebo) as a within-subject and Order (VPA first/placebo first) as a between-subject factor (Figure 4). There was a main effect of Order [*F*_(1, 15)_ = 6.06, *p* = 0.03] due to significantly higher AP scores overall for those who took VPA first. There was also a significant interaction between the factors Condition and Order [*F*_(1, 15)_ = 8.85, *p* = 0.009]. To investigate this interaction, we ran two *post hoc* ANOVAs, one for each treatment order, with Condition (VPA/placebo) as a within-subject factor. For participants who received VPA first, there was a significant effect of Treatment [*F*_(1, 8)_ = 20.25, *p* = 0.002] due to higher scores in the VPA treatment condition compared to placebo. There was no difference between VPA and placebo for the 8 participants who received placebo first [*F*_(1, 7)_ = 1.13, *p* = 0.32].

The ANOVAs over the mood and cognitive measures for the crossover treatment yielded a significant Time X Treatment interaction [*F*_(1, 16)_ = 4.54, *p* = 0.049] for the Altman Self-Rating Mania Scale. Both treatment conditions were associated with a trend toward lower scores post-treatment compared to pre-treatment. However, the decrease from pre- to post-treatment was greater in the VPA condition. A *post hoc* analysis revealed that the change from before to after treatment was significant for VPA (*p* = 0.03, with a Bonferroni correction for multiple comparisons), but not for placebo (*p* = 0.76). We obtained no significant Time X Treatment interaction on any other measure of mood or cognition. Table 3 provides a summary of the results. Except for the RANDOT Stereoacuity test, we conducted an ANOVA with Time (pre-treatment / post-treatment) and Treatment (VPA/placebo) as within-subject factors and Order (VPA first/placebo first) as a between-subject factor. For the ordinal data from the RANDOT Stereoacuity test, we ran a Friedman Test.

**Table 3:** **Analysis of mood and cognitive measures for crossover treatments**.

**Measure**	**VPA first**				**Placebo first**				**Main effects**	**Interactions**
	**VPA**		**Placebo**		**VPA**		**Placebo**			
	**Pre**	**Post**	**Pre**	**Post**	**Pre**	**Post**	**Pre**	**Post**		
**MEASURES OF MOOD**										
**ASRM**	**5.80**	**3.00**	**4.10**	**4.10**	**4.13**	**2.38**	**3.25**	**2.75**		***Time X treatment***
										***F_(1, 16)_ = 4.54, p = 0.049***
BDI II	7.10	7.00	5.10	4.90	6.00	4.00	8.38	8.00		*Treatment X Order*
										*F*(1, 16) = 11.37, *p* = 0.004	
VAS irritable/peaceful	7.86	7.48	7.51	7.29	7.14	7.51	6.84	5.78	*Treatment*: VPA > placebo	
									*F*_(1, 16)_ = 4.89, *p* = 0.04	
VAS interested/disinterested	2.20	2.81	1.70	1.84	3.98	3.18	3.85	3.46	*Order*: VPA first < placebo first	
									*F*_(1, 16)_ = 4.72, *p* = 0.045	
VAS sad	2.18	3.36	1.51	2.42	2.18	2.19	3.43	2.72		*Treatment X order*
										*F*_(1, 16)_ = 8.35, *p* = 0.01
VAS anxious	1.55	2.01	2.40	2.08	2.64	2.41	3.66	3.81	*Treatment*: VPA < placebo	
									*F*_(1, 16)_ = 4.56, *p* = 0.049	
VAS angry	0.72	1.88	1.90	1.77	1.56	1.10	2.51	1.36	*Treatment*: VPA < placebo	*Time X Order*
									*F*_(1, 16)_ = 7.87, *p* = 0.01	*F*_(1, 16)_ = 5.25, *p* = 0.04
VAS happy	6.64	5.85	7.69	6.65	6.61	7.35	6.36	6.71		*Treatment X Order*
										*F*_(1, 16)_ = 4.50, *p* = 0.0498
										*Time X Order*
										*F*_(1, 16)_ = 4.75, *p* = 0.045
**MEASURES OF COGNITION**										
RNBI composite score	22.80	34.60	34.30	27.70	35.62	30.00	22.38	36.50	*Time*: pre > post	*Treatment X Order*
									*F*_(1, 16)_ = 6.76, *p* = 0.02	*F*_(1, 16)_ = 22.75, *p* < 0.001
RAVLT items 1–5	51.50	57.80	50.40	57.40	60.38	61.63	58.75	66.38	*Time*: pre < post	
									*F*_(1, 16)_ = 32.43, *p* < 0.001	
VAS mentally slow/quick-witted	7.31	5.99	7.23	6.68	4.91	6.20	4.84	6.03		*Time X Order*
										*F*_(1, 16)_ = 6.00, *p* = 0.03
VAS alert/drowsy	4.07	3.33	2.33	2.99	5.38	4.72	5.41	4.86	*Order*: VPA first < placebo first	
									*F*_(1, 16)_ = 5.02, *p* = 0.04	
Stroop color-word	43.80	46.40	50.60	53.40	54.62	54.50	48.25	52.38		*Treatment X Order*
										*F*_(1, 16)_ = 27.59, *p* < 0.001
Regan visual acuity (Left Eye)	1.10	2.70	2.70	2.10	1.75	2.25	2.38	1.25		*Treatment X Order*
										*F*_(1, 16)_ = 8.10, *p* = 0.01

*The only significant Time x Treatment interaction, indicating a differential effect of VPA on measures of mood and cognition pre- and post-treatment, is highlighted in bold*.

The experiment was double-blind, as neither participants, nor experimenters knew the randomization for treatment conditions. However, we did ask participants to intuit in which arm they received VPA treatment, and why they thought so. We also instructed them to write down any side effects they experienced during the experiment. Out of the 18 participants who completed the second treatment arm, 17 guessed correctly. A small number of them said they felt they were functioning at a higher cognitive level when taking what they thought was the VPA, but most said that mild side effects like drowsiness and nausea were the primary cues that they used to determine which was the active treatment. It needs to be noted, however, that participants' opinion about the drug taken was unlikely to influence the results for at least two reasons. First, participants could guess, but could not be sure about the substance taken. Second, they were naïve with respect to the hypothesis tested and could thus not voluntarily influence their behavior in the expected direction.

## Discussion

Until now we had no mechanistic account of the neural processes underlying the critical period of AP. More generally, we have lacked human experimental models with which to measure the potential for a compound to facilitate neuroplasticity in the adult human brain. This study provides the “proof-of-concept” for the possibility to restore neuroplasticity using a drug by offering evidence for a possible effect of VPA on AP perception. In confirmation of our hypothesis, AP performance varied according to treatment condition. Normal male volunteers performed significantly better on a test of AP after 2 weeks of VPA treatment than after 2 weeks of placebo.

Certain aspects of our findings warrant further discussion. First, it was not possible to establish baseline performance on the AP task, as the association between the musical notes and the names was necessarily established during training. One possible option would have been to test participants' AP performance early in the regimen, while they were gradually reaching the full dose of VPA. We had decided not to implement such a baseline test, as no precise information was available regarding the time course of the effect of VPA under the current conditions, so AP training was only started on day 8, once participants have reached the full dose. The absence of a baseline test might introduce ambiguity into the interpretation of the effect. Indeed, there are identifiable individual differences in musical ability, and in the neuroanatomical structures supporting it (see Herholz and Zatorre, 2012 for a review), which might have led to participants with better AP abilities assigned to the VPA group in the first treatment arm by chance. However, there is evidence in our study that the significant effect of treatment for the AP task cannot be fully accounted for by a between-group difference in the ability to acquire AP. If such a difference were the driving force, then we would have expected that in the second treatment arm the participants in the potentially high-ability group (i.e., those who were in the VPA condition for the first treatment arm) would outperform the low-ability group (i.e., those who were in the placebo condition for the first treatment arm). However, this was not the case. Further, since our participants were musically naïve (participants who received musical training all started after the age of 7 years, with a mean of 12 years), the presence of complete or partial AP possessors in either of the groups is very unlikely. As reported above, there was no significant difference between the two groups in the second treatment arm. Further, all of the top scorers from the VPA condition in the first treatment arm completed the second treatment arm, eliminating the possibility that the participants who completed the second treatment arm were not representative of the group from the first treatment arm. This strengthens our interpretation that the VPA treatment led to the significantly higher performance in the VPA condition compared to placebo.

Second, the analysis of the crossover, i.e., of the 17 participants for whom we have data from both arms, revealed an order-dependent effect of treatment. For participants who took VPA first, AP performance was significantly higher after VPA treatment than after placebo. In contrast, for participants who initially took placebo, there was no such difference. It may be that carry-over effects impeded performance on the AP task in the second treatment arm. A memory conflict between the pitch classes and proper names used in the first treatment arm could have interfered with those used in the second. Because of this order effect, the most reliable comparison focused on the first treatment arm. Future research should aim to use a randomized controlled trial (RCT) design with treatment condition as a between-participants factor. Doing so would avoid any possible carry-over effects from one treatment arm to the next. Relatedly, it needs to be noted that we did not test how long the effect of the improvement in AP perception lasted. Future research will need to address this question by retesting participants after several days or weeks following the end of training.

Third, our study was not set up to measure reaction times. Participants were informed that they had 4 s available for their response with no instruction to do so rapidly. However, as AP possessors are known to recognize pitch classes faster than non-AP possessors do (Levitin and Rogers, 2005), reaction times constitute a relevant measure to use in future follow-up studies.

Fourth, the sizes of the groups tested were relatively small compared to some recent AP studies, which might explain why no significant correlation was observed between AP performance and training compliance, i.e., the amount of training received. However, when compared to studies using VPA with healthy participants, our sample sizes were quite appropriate (e.g., *n* = 12 in the placebo as well as in the VPA group in the (Bell et al., 2005a,b) study).

Fifth, we observed an effect of VPA on the Altman Self-Rating Mania Scale. Indeed, VPA is commonly used as a mood stabilizer to control mania in bipolar disorder (Macritchie et al., 2001). Our results in healthy participants therefore confirmed the clinical action of VPA, which may have an effect on sub-clinical levels of mania as well. Importantly, however, an improved or more stable mood in the VPA condition cannot explain the obtained results, as we would then expect general cognitive improvement in the other neurocognitive tasks administered, as well as greater AP performance in both treatment arms.

In sum, our study is the first to show a change in AP with any kind of drug treatment. The finding that VPA can restore plasticity in a fundamental perceptual system in adulthood provides compelling evidence that one of the modes of action for VPA in psychiatric treatment may be to facilitate reorganization and rewiring of otherwise firmly established pathways in the brain and its epigenome (Shen et al., 2008).

Valproic acid is believed to have multiple pharmacological actions, including acute blockade of GABA transaminase to enhance inhibitory function in epileptic seizures and enduring effects on gene transcription as an histone deacetlyase (HDAC) inhibitor (Monti et al., 2009). Of relevance here is the epigenetic actions of this drug, as enhancing inhibition does not reactivate brain plasticity in adulthood (Fagiolini and Hensch, 2000), but reopening chromatin structure does (Putignano et al., 2007). While systemic drug application is a rather coarse treatment, the effects may differ dramatically by individual cell type (TK Hensch and P Carninci, unpublished observations). VPA treatment mimics Nogo receptor deletion to reopen plasticity for acoustic preference in mice (Yang et al., 2012), suggesting a common pathway through the regulation of myelin-related signaling which normally closes critical period plasticity (McGee et al., 2005). Future work will address the cellular actions of VPA treatment in the process of reactivating critical periods. Future MRI studies will also be needed to establish whether HDAC inhibition by VPA induces hyperconnectivity of myelinated, long-range connections concurrent with renewed AP ability (Loui et al., 2011).

If confirmed by future replications, our study will provide a behavioral paradigm for the assessment of the potential of psychiatric drugs to induce plasticity. In particular, the AP task may be useful as a behavioral correlate. If further studies continue to reveal specificity of VPA to the AP task (or to tasks on which training or intervention is provided), critical information will have been garnered concerning when systemic drug treatments may safely be used to reopen neural plasticity in a specific, targeted way.

### Conflict of interest statement

The authors declare that the research was conducted in the absence of any commercial or financial relationships that could be construed as a potential conflict of interest.

## Footnotes

^1^Along with the adult test of AP reported here, participants were also tested on pilot versions of infant perceptual tasks, which we are developing for use with adults.

^2^A software malfunction corrupted the data for one participant in the first treatment arm, leaving 11 who took VPA and 12 who took placebo.
